# The Impact of Epstein-Barr Virus Infection on Juvenile Idiopathic Arthritis Activity and Patient’s Response to Treatment

**DOI:** 10.3390/jcm9113453

**Published:** 2020-10-27

**Authors:** Violetta Opoka-Winiarska, Ewelina Grywalska, Aleksandra Sobiesiak, Jacek Roliński

**Affiliations:** 1 Department of Paediatric Pulmonology and Rheumatology, Medical University of Lublin, 20-093 Lublin, Poland; ola6891@wp.pl; 2 Department of Clinical Immunology and Immunotherapy, Medical University of Lublin, 20-093 Lublin, Poland; ewelina.grywalska@gmail.com (E.G.); jacek.rolinski@gmail.com (J.R.)

**Keywords:** EBV, juvenile idiopathic arthritis, arthritis

## Abstract

This study aimed to investigate the relationship between Epstein-Barr virus (EBV) infection and the onset of juvenile idiopathic arthritis (JIA), disease activity, and response to treatment. The study included 44 children with JIA, 23 children with different types of arthritis, and 44 controls. We measured EBV infection markers, including the EBV DNA load and the concentration of antibodies to viral antigens, at disease onset, before treatment. Six months after JIA diagnosis and the initiation of treatment patients with anti-viral capsid antigen IgG had a higher disease activity and worse response to treatment than patients without previous infection. After six months of treatment, the probability of disease inactivity in children without a history of EBV infection was almost 6.5 times greater than in a child with a history of infection. Furthermore, the probability of a better response after six months of treatment in a child with a history of EBV infection was more than five times smaller than in a child without infection. A past EBV infection can have a negative effect on achieving disease remission and may be associated with a worse response to treatment. Our results do not indicate the need for routine assessment of EBV infection markers in patients with JIA.

## 1. Introduction

Juvenile idiopathic arthritis (JIA) is the most common chronic rheumatic childhood disease of still unknown aetiology [1]. Apart from genetic factors, environmental factors play a significant role in the pathogenesis of the disease [1,2,3].

Modern pharmacological therapies enable effective control of the disease, but many JIA patients experience exacerbations during therapy or do not respond to medications [1,3]. Therefore, new data on the pathogenesis of JIA may allow for more effective interventions.

One of the factors that may influence the development of the disease is infection by the Epstein-Barr virus (EBV). However, it has not yet been clarified whether EBV infection may be related to disease activity or patient response to treatment.

The objective of this study was to define the relationship between EBV infection and the onset of JIA, disease activity, and patient response to treatment.

## 2. Experimental Section

### 2.1. Patients

The study was conducted in three groups of children: 44 patients with newly diagnosed JIA according to International League of Associations for Rheumatology (ILAR) criteria, previously untreated [4], 23 patients with different types of arthritis (reactive, infectious, but not septic) also newly diagnosed, and 44 children (without inflammatory, autoimmune diseases and infections) participating as controls.

The JIA group included children with oligoarthritis (21), polyarthritis with positive RF (2) polyarthritis with negative RF (13), enthesitis related arthritis (7), psoriatic arthritis (1).

All patients included in the study were diagnosed and treated in the Department of Paediatric Pulmonology and Rheumatology, Medical University of Lublin. Children with systemic JIA were excluded from the study due to the small number of patients and the otherness of pathogenesis and course.

We quantified EBV infection markers in all the groups, including EBV DNA load in peripheral blood mononuclear cells (PBMC) and serum concentration of immunoglobulin (Ig) A, G, and M directed to the viral capsid antigen (VCA), EBV early antigen (EA), and nuclear antigen 1 (EBNA-1). In the groups with arthritis, these tests were performed after diagnosis but before treatment with disease-modifying antirheumatic drugs (DMARDs) or glucocorticoids.

Inflammatory markers, including the erythrocyte sedimentation rate (ESR) and C-reactive protein (CRP) were examined during routine outpatient visits.

In patients with JIA, the disease activity was estimated with the use of the Juvenile Arthritis Disease Activity Score 71 (JADAS 71) [5] on the day of diagnosis (point zero) and after three and six months. The response to treatment was estimated according to the American College of Rheumatology Pediatric response criteria (PedACR) [5] after three and six months of treatment. In all JIA patients, at least one DMARD was used during the first six months after diagnosis: methotrexate, sulfasalazine, or both. Methotrexate (at a dose of 10–15 mg/m^2^ body surface area per week administered orally or subcutaneously) as a single drug or in combination with another DMARD was prescribed to 37 (84.1%) patients from diagnosis up to three months and to 39 (88.6%) patients from three to six months. During the therapy, the dosage and route of methotrexate administration were modified at physician’s discretion, depending on the disease activity and treatment tolerance. In all treated patients the therapy was continued during the six-month follow-up period. No patient was treated with biological DMARDs during this period. No patient and no control child had symptoms of a new EBV infection during the six-month follow-up.

### 2.2. Sample Preparation

We collected 7 mL of peripheral blood in EDTA-coated tubes (Sarstedt, Nümbrecht, Germany). We isolated PBMC and plasma using density gradient centrifugation (400 g, 30 min, no brake). Briefly, 5 mL of whole blood was diluted with 5 mL of saline and was applied on 5 mL of Ficoll-Paque™ (Miltenyi Biotec, Bergisch-Gladbach, Germany). The PBMC layer was harvested, the cells were counted, and viability was assessed with the trypan blue exclusion method (0.4% trypan blue solution; Sigma Aldrich, Hamburg, Germany). Only PBMC samples with a viability ≥95% were used. Plasma was stored at −80 °C until further use.

### 2.3. DNA Isolation, Calculation of EBV Load, and Assessment of Anti-EBV Antibody Status

DNA was isolated from PBMC using the QIAamp DNA Blood Mini Kit (QIAGEN, Hilden, Germany) according to the manufacturer’s instructions. The number of EBV-specific DNA copies in PBMC was calculated with the ISEX version of the EBV polymerase chain reaction (PCR) kit (GeneProof, Brno, Czech Republic). A specific conservative DNA sequence for the EBNA-1 gene was amplified using real-time PCR. The number of viral DNA copies per μL of eluent was adjusted for the efficiency of DNA isolation, to be expressed as the number of viral DNA copies per μg of DNA. All samples were examined in duplicate. A corresponding negative control (DNA elution buffer) was also included. The concentration and purity of the isolated DNA were verified with a BioSpec-nano spectrophotometer (Shimadzu, Kyoto, Japan). All samples below 10 DNA copies per μL were considered EBV negative because of the detection threshold of the spectrophotometer. A 7300 Real Time PCR System (Applied Biosystems, Foster City, CA, USA) was used for the analyses.

EBV-specific Abs were quantified with a commercial enzyme-linked immunosorbent assays (ELISA; IBL International, Hamburg, Germany), using an ELISA Reader Victor TM3 (PerkinElmer, Waltham, MA, USA). IgA, IgM, and IgG antibody classes recognizing EBV antigens: EA, VCA, and EBNA-1 were measured. Manufacturer-specified cut-offs were applied.

### 2.4. Statistical Analysis

Statistical methods were used to estimate the dependencies between the EBV infection and the development of arthritis, disease activity and the response to treatment. The statistical analysis was performed with StatSoft, Poland Statistica v. 10.0 software. Categorical variables are expressed as the number of observations and percentage, whereas continuous variables are shown as means, standard deviations, medians, and first and third quartiles. Because the distributions of data were skewed (assessed with Shapiro–Wilk W test) or variances were heterogenous (assessed with Fisher’s F test), nonparametric statistics were applied to analyze differences between groups. For the comparison of two groups, Mann–Whitney’s U test was used, and for comparison of three or more subgroups, the Kruskal–Wallis H test followed by multiple-comparison post-hoc test was used. The Spearman R coefficient was used to analyze correlations between variables. For the analysis of categorical variables with a small number of observations (*n* < 5), the χ^2^ test with Yates correction was applied. Furthermore, *p* values less than 0.05 were considered significant.

### 2.5. Compliance with Research Ethics Standards

All patients and parents/legal guardians were informed in detail in oral and written form about the course, aims, and scope of the conducted research and signed an informed written consent to participate in the study. The study was carried out in compliance with the Declaration of Helsinki. The study design was approved by the Bioethics Committee at the Medical University of Lublin (KE-0254/263/2015).

## 3. Results

### 3.1. Baseline Characteristics of the Patients

Baseline demographic and clinical characteristics of the studied groups are shown in Table 1.

### 3.2. Relationship Between EBV Infection and the Onset of JIA

There were no statistically significant differences in the serum concentrations of anti-EBV Abs between the JIA group and the group with other types of arthritis and between the JIA and control group (*p* > 0.05; Table 2). Similarly, there were no significant differences in the EBV DNA load between these groups (*p* > 0.05; Table 2).

Comparisons between patients with different JIA types and the control group did not reveal any significant differences (*p* > 0.05; Table 3).

Results were considered positive when the Abs concentration exceeded the following thresholds: >1.1 U/mL anti-VCA, >1.2 U/mL anti-EA, and >1.2 U/mL anti-EBNA-1 (Table 4).

To assess whether the history of EBV infection is associated with the onset of JIA or other types of arthritis, the percentage of patients with positive anti-VCA IgG (>1.1 U/mL) was compared in the three study groups (Table 4). The logistic regression model revealed no impact of past EBV infections on the prevalence of any type of arthritis (*p* = 0.46) (Table 5).

To assess whether EBV viremia is associated with the onset of JIA or other types of arthritis, the percentage of patients with a positive viral load was compared in the three study groups (Table 4). The logistic regression model revealed no impact of active EBV infection on the prevalence of any type of arthritis (*p* = 0.62) (Table 5).

### 3.3. Relationship between EBV Infection and JIA Activity

To assess whether the history of EBV infection affects disease activity in JIA, the JADAS 71 score was compared between patients that were positive and negative for anti-VCA IgG Abs (Table 6).

The prevalence of positive anti-VCA IgG was associated with disease activity at month 6 of the disease (*p* = 0.04, Table 6). Therefore, we evaluated the presence of antibody in patients with inactive (JADAS 71 < 1) and active (JADAS 71 ≥ 1) disease at three and six months from diagnosis (Table 7).

The logistic regression model showed a significantly higher occurrence of positive anti-VCA IgG results in patients with active disease than in patients with inactive disease at six months from diagnosis. The probability of disease inactivity after six months of treatment in a child without a history of EBV infection was almost 6.5 times greater than in a child with EBV infection (odds ratio [OR] = 6.4; 95% CI: 1.1–37.8).

Significantly higher disease activity at six months after diagnosis was demonstrated in patients with positive anti-VCA IgG results than in those without evidence of previous infection (*p* = 0.04; Table 6).

To assess whether EBV viremia affects disease activity in JIA, the JADAS 71 score was compared in JIA patients with positive and negative EBV DNA results (Table 6). No significant differences were found between the groups.

### 3.4. Relationship between EBV Infection and Response to Treatment in JIA Patients

To assess whether the history of EBV infection affects the response to treatment in the course of JIA, PedACR was compared in patients with positive (>1.1 U/mL) and negative (≤1.1 U/mL) anti-VCA IgG results (Table 8).

The prevalence of positive anti-VCA IgG was associated with PedACR at month 6 of the disease (*p* = 0.049; Table 8) Therefore, we evaluated the presence of Ab in patients with a poor (PedACR 30/50) and good (PedACR 70/90) response after three and six months of treatment (Table 9).

Patients with positive anti-VCA IgG results had a significantly worse response to treatment after six months than those without evidence of previous infection (*p* = 0.049; Table 8).

The logistic regression model showed a significantly higher occurrence of positive anti-VCA IgG results in patients with poor response to treatment than in patients with good response at six months from diagnosis. The probability of having a better response to treatment after six months of treatment in a child with a history of EBV infection was more than five times lower than in a child without an infection (OR = 0.20; 95% CI: 0.03–1.18).

To assess whether EBV viremia affects the response to treatment, the PedACR scores were compared in JIA patients with positive and negative results for EBV DNA (Table 8) after three and six months of treatment. No significant differences were found.

## 4. Discussion

In this study we sought to determine the significance of EBV infection in the pathogenesis of JIA by assessing markers of EBV infection in patients with a newly diagnosed arthritis, previously untreated: JIA, other types arthritis, and controls.

We did not observe any effect of past EBV infections on arthritis prevalence or any correlation between EBV infection and the onset of JIA.

There are only a limited number of published reports regarding the occurrence of Abs to EBV antigens in patients with JIA, [6,7,8,9] and the conclusions they reach are unclear.

Gear et al. [8] examined the occurrence of anti-VCA IgG in patients with JIA and found an incidence of Abs in the entire population that was similar to that in our study (43.1%). Their data did not confirm the role of EBV infection in the pathogenesis of JIA, which is consistent with the results of our study.

Tsai et al. [9] compared anti-EBV IgG concentrations in children with JIA, lupus erythematosus, and controls and found no significant differences. They suggest that immunodeficiency may be contributing to the increased susceptibility to EBV infection in children with rheumatic diseases.

Aghighi et al. [6] assessed the presence of anti-VCA IgM and IgG in Iranian children with newly diagnosed JIA and found a similar (44%) percentage of patients with IgG Abs as in our study. They observed a high percentage of children with IgM Abs (82%) corresponding to the frequency of EBV infection in JIA. The authors concluded that, considering the data on healthy children (70%), this indicated a relationship between EBV infection and JIA pathogenesis [6]. However, doubts over their interpretation result from the high frequency of anti-VCA IgM Abs (82% of patients with JIA) and the fact that no signs of infection were reported in any of the patients.

Published studies to date have not revealed differences in the number of EBV copies in the PBMC of patients with JIA compared to controls [9,10,11,12]. In our study, we did not observe any differences in the occurrence of EBV viremia in the studied groups, so the connection with the prevalence of arthritis was not demonstrated.

In summary, our study did not show that EBV infection was significant in inducing an immune disorder leading to an uncontrolled inflammatory process that results in JIA symptoms.

To assess whether the previous EBV infection affects disease activity in JIA, JADAS 71 [5] was compared in patients with positive and negative anti-VCA IgG results. The evaluation the response to treatment after six months of therapy according to American College of Rheumatology Pediatric Criteria (PedACR) showed that the probability of a better response to treatment for a child with a history of EBV infection was more than five times smaller than for a child without previous EBV infection.

We observed also significantly higher anti-VCA IgG concentrations in children with active disease than in children with inactive disease six months after diagnosis. Furthermore, the chance of disease inactivity after 6 months of treatment in a child without a history of EBV infection was almost 6.5 times greater than those with evidence of previous EBV infection.

Our study has therefore shown that past EBV infection can be important for long-term disease activity and response to treatment. We did not observe association between EBV viral load and response to treatment.

Aghighi et al. [6] evaluated the response to treatment after six months of therapy according to the presence of IgM or IgG against the EBV VCA antigen at the time of diagnosis. The response was defined as good in case of continuing conventional therapy or poor, in case of exacerbation. In a group of 50 patients with JIA, a poor response was found in 50% of patients with anti-VCA IgG and 12% without these antibodies and in 22% with anti-VCA IgM and 40% without these antibodies. Like in our study, the authors conclude that past EBV infection is a factor that affects the response to treatment in JIA patients [6].

In summary, our study showed that past EBV infection in JIA patients may be associated with higher disease activity and poorer response to treatment during the first six months of the disease. Pre-treatment of EBV viremia was not relevant for disease activity and response to treatment.

So far, little data on the effect of EBV on JIA activity and response to treatment [6], but recent publications indicate an association between EBV and activity of juvenile systemic lupus erythematosus [13]. These results suggest that EBV contributes to disease continuity, even if it does not directly cause development.

The recurrent course of JIA is considered as related to dysfunction of T regulatory (T reg) cells, which are responsible for suppressing inflammation and the development of autoimmunity. T-helper (Th) 17 cells were identified also as key cells in the joints of patients with JIA, however whole group of T-cell subsets are involved in unstable inflammatory process [3].

This may be related to the recent data indicates the involvement of EBV in inflammatory reactions via the modulation of two major cell compartments: Th17 cells which contribute to the development of inflammation and autoimmunity by producing IL-17A, and T reg cell activities [14].

To explain how EBV affects JIA activity and response to treatment, we hypothesized that the virus might operate by epigenetic mechanisms.

Epigenetic modifications such as DNA methylation, histone modification, and noncoding RNA translate environmental stimuli to switch on and off gene expression and therefore regulate cell function [15]. EBV might induce epigenetic changes in host cells, via DNA methylation and histone modifications. The viruses can manipulate epigenetic processes to influence host responses associated with immunity and inflammation [16]. Altered DNA methylation has been associated with adult autoimmune rheumatic diseases such as rheumatoid arthritis [17]. Studies in patients with rheumatoid arthritis [17] have shown epigenetic changes in synovial fibroblasts (main stromal cells of the joint synovium) associated with activation of these cells. The methylation changes might involve in the course of arthritis and response to therapeutics [15].

## 5. Conclusions

Our study did not confirm the relationship between EBV viremia or past EBV infection and the onset of JIA.

A past EBV infection can have a negative effect on achieving disease remission, and it may be associated with a worse response to treatment.

Our results do not indicate the need for routine assessment of EBV infection markers in patients with JIA. The examination should be recommended in case of persistent disease activity or lack of an expected response to treatment.

## Figures and Tables

**Table 1:** Baseline demographic and clinical characteristics of the patients.

Characteristic	JIA (*n* = 44)	Other Types of Arthritis (*n* = 23)	Control (*n* = 44)
Age, years	8.9 ± 4.5	11.9 ± 4.5	10.8 ± 3.9
Age, years (median)	8	13	12
Sex, female (%)	31 (70%)	14 (61%)	29 (66%)
JIA type, *n* (%)			
Oligoarthritis	21 (48%)	-	-
Polyarthritis RF+ Polyarthritis RF−	2 (4%) 13 (930%)	- -	- -
ERA Psoriatic arthritis	7 (16%) 1 (2%)	- -	- -
WBC (×10^3^/µL)	8.4 ± 2.7	8.3 ± 2.5	6.6 ± 1.8
RBC (×10^6^/µL)	4.7 ± 0.3	4.6 ± 0.5	4.6 ± 0.3
HGB (g/dL)	12.6 ± 1.2	13.0 ± 1.6	13.2 ± 1.1
PLT (×10^3^/µL)	355 ± 130	313 ± 88	284 ± 70
CRP (mg/dL)	0.54 ± 1.15	2.19 ± 2.88	0.12 ± 0.31
ESR (mm/h)	29.8 ± 25.1	31.6 ± 29.6	7.4 ± 7.6
LAS	5.6 ± 6.5	1.7 ± 1.3	0 ± 0
LORS	2.9 ± 5.3	0.74 ± 1.0	0 ± 0
VAS Ph (cm)	5.2 ± 2.1	ND	ND
VAS P (cm)	5.7 ± 2.1	ND	ND
CHAQ	0.85 ± 5.60	ND	ND

Values are shown as means ± standard deviation, unless stated otherwise. JIA, juvenile idiopathic arthritis; ERA, enthesitis-related arthritis; RF+—rheumatoid factor positive; RF−—rheumatoid factor negative, WBC, white blood cells; RBC, red blood cells; HGB, hemoglobin; PLT, platelet count; CRP, C-reactive protein; ESR, erythrocyte sedimentation rate; AJC, Active joint count; ROM—joints with limited range of motion; VAS Ph, physician global assessment of disease activity (0–10 cm visual analog scale); VAS P, parent/patient assessment of overall well-being (0–10 cm VAS); CHAQ, Childhood Health Assessment Questionnaire; ND, not determined.

**Table 2:** Serum concentrations of EBV infection markers in patients with JIA, other types of arthritis, and controls. Results are presented as mean (median) ± standard deviation.

EBV Infection Marker		JIA (*n* = 44)	Other Types of Arthritis (*n* = 23)	Control (*n* = 44)	*p*-Value
EA (U/mL)	IgM	0.69 (0.54) ± 0.38	0.66 (0.55) ± 0.32	0.68 (0.64) ± 0.34	0.90
	IgA	0.33 (0.32) ± 0.14	0.42 (0.34) ± 0.23	0.34 (0.29) ± 0.20	0.23
	IgG	0.23 (0.19) ± 0.15	0.29 (0.17) ± 0.48	0.20 (0.14) ± 0.17	0.80
EBNA-1 (U/mL)	IgA	0.11 (0.1) ± 0.05	0.13 (0.11) ± 0.06	0.10 (0.07) ± 0.06	0.10
	IgG	0.46 (0.51) ± 0.29	0.53 (0.66) ± 0.26	0.46 (0.44) ± 0.31	0.53
	IgM	0.19 (0.16) ± 0.08	0.23 (0.16) ± 0.18	0.20 (0.17) ± 0.13	0.87
VCA (U/mL)	IgA	0.23 (0.17) ± 0.22	0.26 (0.19) ± 0.20	0.24 (0.17) ± 0.21	0.40
	IgG	0.96 (1.04) ± 0.51	0.67 (0.53) ± 0.57	0.91 (0.95) ± 0.54	0.11
Viral load	Copy number/µL	0.34 (0) ± 0.74	2.14 (0) ± 5.92	0.48 (0) ± 0.81	0.65
	Copy number/µg DNA	3.19 (0.00) ± 6.78	21.03 (0) ± 60.36	4.77 (0.00) ± 7.94	0.62
	Copy number/100,000 cells	2.10 (0.00) ± 4.48	13.88 (0.00) ± 39.84	3.15 (0.00) ± 5.24	0.62
	DNA ng/µL	101.13 (90.59) ± 32.90	102.8 (102.69) ± 35.35	94.98 (89.61) ± 29.23	0.76

EA—early antigen; EBNA-1—Epstein-Barr nuclear antigen1; JIA—juvenile idiopathic arthritis; VCA—viral capsid antigen.

**Table 3:** Serum concentrations of infection markers in patients with different types of JIA and controls. Results are presented as mean (median) ± standard deviation.

EBV Infection Marker		JIA (*n* = 44)			Control (*n* = 44)	*p*-Value
		Oligoarthritis (*n* = 21)	Polyarthritis RF+ & RF− (*n* = 15)	ERA & Psoriatic Arthritis (*n* = 8)		
EA (U/mL)	IgM	0.63 (0.50) ± 0.31	0.76 (0.72) ± 0.35	0.73 (0.46) ± 0.58	0.68 (0.64) ± 0.34	0.67
	IgA	0.37 (0.39) ± 0.16	0.31 (0.3) ± 0.12	0.26 (0.27) ± 0.10	0.34 (0.29) ± 0.20	0.16
	IgG	0.24 (0.19) ± 0.17	0.23 (0.18) ± 0.15	0.17 (017) ± 0.05	0.2 (0.14) ± 0.17	0.20
EBNA-1 (U/mL)	IgA	0.11 (0.09) ± 0.05	0.11 (0.09) ± 0.05	0.10 (0.1) ± 0.04	0.10 (0.07) ± 0.06	0.10
	IgG	0.42 (0.43) ± 0.28	0.57 (0.67) ± 0.28	0.36 (0.27) ± 0.31	0.46 (0.44) ± 0.31	0.27
	IgM	0.18 (0.15) ± 0.07	0.20 (0.16) ± 0.08	0.19 (0.17) ± 0.10	0.20 (0.17) ± 0.13	0.20
VCA (U/mL)	IgA	0.23 (0.17) ± 0.19	0.20 (0.18) ± 0.13	0.29 (0.12) ± 0.40	0.24 (0.17) ± 0.21	0.69
	IgG	1.03 (1.09) ± 0.50	0.10 (0.98 ± 0.54	0.87 (0.85) ± 0.51	0.91 (0.95) ± 0.54	0.27
Viral load	Copy number/µL	0.37 (0) ± 0.67	0.16 (0) ± 0.57	0.58 (0) ± 0.16	0.48 (0) ± 0.81	0.64
	Copy number/µg DNA	4.37 (0) ± 7.97	1.00 (0) ± 3.38	4.19 (0) ± 7.91	4.77 (0) ± 7.94	0.59
	Copy number/100,000 cells	2.28 (0) ± 5.26	0.66 (0) ± 2.23	2.77 (0) ± 5.22	3.15 (0) ± 5.24	0.59
	DNA ng/µL	90.49 (84.8) ± 28.34	104.09 (90.3) ± 34.14	123.53 (126.69) ± 33.07	94.98 (89.61) ± 29.23	0.25

EA—early antigen; EBNA-1—Epstein-Barr nuclear antigen 1; ERA—enthesitis-related arthritis; JIA—juvenile idiopathic arthritis; RF+—rheumatoid factor positive; RF−—rheumatoid factor negative; VCA—viral capsid antigen.

**Table 4:** Frequency of positive results of anti-EBV antibodies and positive viral load results in patients with JIA, other types of arthritis, and controls.

EBV Infection Marker		JIA (*n* = 44) *n* (%)	Other Types of Arthritis (*n* = 23) *n* (%)	Control (*n* = 44) *n* (%)
VCA	IgA	1 (2.3)	0	0
	IgG	19 (43.1)	7 (30.4)	20 (45.4)
EA	IgM	5 (11.4)	3 (13.0)	4 (9.1)
	IgA	0	0	1 (2.3)
	IgG	0	1 (4.3)	0
EBNA-1	IgA	0	0	0
	IgG	0	0	1 (2.3)
	IgM	0	0	0
Positive viral load results		11 (25%)	6 (26%)	8 (18%)
Range (min-max)	copy number/µL	0–3.35	0–21.67	0–2.84
	copy number/µg DNA	0–23.06	0–268.16	0–23.26
	copy number/100,000 cells	0–15.22	0–176.98	0–15.35
	concentration DNA ng/µL	0–171.16	0–170.88	0–192.7

EA—early antigen; EBNA-1—Epstein-Barr nuclear antigen 1; EBV—Epstein-Barr virus; JIA—juvenile idiopathic arthritis; VCA—viral capsid antigen.

**Table 5:** Prevalence of positive anti-VCA IgG (>1.1 U/mL) and viral load test in patients with JIA, other types of arthritis, and controls; CI—confidence interval.

Group of Patients	% VCA IgG >1.1 (95% CI)	% Positive of Viral Load (95% CI)
JIA (*n* = 44)	43.1% (28.3–59.0%)	25% (13.2–40.3%)
Other types of arthritis (*n* = 23)	30.4% (13.2–52.9%)	25.1% (10.2–48.4%)
Control (*n* = 44)	45.5% (30.4–61.2%)	36.4% (17.2–59.3%)
Total (*n* = 111)	41.4% (32.2–51.2%)	28.1% (19.1–38.6%)

JIA—juvenile idiopathic arthritis; VCA—viral capsid antigen.

**Table 6:** Comparison of disease activity (JADAS 71) in JIA patients according to anti-VCA IgG concentration (positive > 1.1 vs. negative ≤ 1.1 U/mL) and EBV DNA results at the time of diagnosis and after 3 and 6 months. Results are presented as mean (median) ± standard deviation.

	JADAS 71 According to Anti-VCA IgG Concentration Status			JADAS 71 According to EBV DNA Status		
Time from Diagnosis	Positive *n* = 11	Negative *n* = 33	*p*-Value	Positive *n* = 11	Negative *n* = 33	*p*-Value
0	17.30 (18.0) ± 9.36	18.08 (17.0) ± 10.29	0.77	15.75 (10.0) ± 9.57	18.41 (19.0) ± 9.92	0.35
3 months	8.59 (5.0) ± 9.17	7.32 (6.0) ± 6.83	0.87	5.71 (6.0) ± 6.38	8.85 (6.0) ± 8.26	0.32
6 months	4.18 (1.0) ± 5.03	1.49 (0) ± 3.19	0.04	2.31 (0) ± 3.57	2.8 (0) ± 4.54	0.86

JADAS—juvenile arthritis disease activity score; VCA—viral capsid antigen.

**Table 7:** Prevalence of positive anti-VCA IgG results (>1.1 U/mL) in patients with inactive (JADAS 71 < 1) and active (JADAS 71 ≥ 1) disease; CI—confidence interval.

Group	Patients with Positive Anti-VCA IgG Concentration (%) (95% CI)
JADAS 71 < 1 (*n* = 34)	35.3% (13.2–40.3%)
JADAS 71 ≥ 1 (*n* = 10)	77.8% (10.2–48.4%)
Total (*n* = 44)	43.1% (19.1–38.6%)

JADAS—juvenile arthritis disease activity score; VCA—viral capsid antigen.

**Table 8:** Comparison of the response to treatment (PedACR) in JIA patients according to anti-VCA IgG concentration (positive > 1.1 vs. negative ≤ 1.1 U/mL) and EBV DNA results after 3 and 6 months of treatment. Results are presented as mean (median) ± standard deviation.

	PedACR Score According to Anti-VCA IgG Results			PedACR Score According EBV DNA Results		
Months of treatment	Positive *n* = 11	Negative *n* = 33	*p*-value	Positive *n* = 11	Negative *n* = 33	*p*-value
3	56.31 (50.00) ± 22.16	58.0 (50.00) ± 25.17	0.91	62.73 (50.00) ± 24.12	55.45 (50.00) ± 23.6	0.37
6	73.16 (90.00) ± 22.31	85.0 (90.00) ± 12.16	0.049	75.45 (90.00) ± 22.07	81.21 (90.00) ± 16.8	0.43

EBV—Epstein-Barr virus; PedACR—American College of Rheumatology Pediatric Criteria; VCA—viral capsid antigen.

**Table 9:** Prevalence of positive anti-VCA IgG (>1.1 U/mL) results after six months of treatment in patients with a poor (PedACR 30/50) and good (PedACR 70/90) response to treatment; CI—confidence interval.

Group	Patients with Positive Anti-VCA IgG Results (%) (95% CI)
PedACR 30/50 *n* = 9	75.0% (34.9–96.8%)
PedACR 70/90 *n* = 35	37.1% (21.5–55.1%)
Total (*n* = 44)	43.1% (32.2–51.2%)

PedACR—American College of Rheumatology Pediatric Criteria; VCA—viral capsid antigen.
